# Planning for an uncertain future in progressive neurological disease: a qualitative study of patient and family decision-making with a focus on eating and drinking

**DOI:** 10.1186/s12883-018-1112-6

**Published:** 2018-08-16

**Authors:** Gemma Clarke, Elizabeth Fistein, Anthony Holland, Jake Tobin, Sam Barclay, Stephen Barclay

**Affiliations:** 1 Primary Care Unit, Department of Public Health and Primary Care, Cambridge Institute of Public Health, University of Cambridge School of Clinical Medicine, Box 113 Cambridge Biomedical Campus, Cambridge, CB2 0SR UK; 2 The Health Foundration, Chair in Learning Disabilities, Cambridge Intellectual and Developmental Disabilities Research Group, Department of Psychiatry, University of Cambridge, Cambridge, UK; 3 University of Cambridge, Cambridge, UK

**Keywords:** Decision-making capacity, Dysphagia, Care planning, Mealtimes, Dementia, Parkinson’s disease, Huntington’s disease, Progressive Supranuclear palsy, Motor Neurone disease, Multiple sclerosis

## Abstract

**Background:**

Dysphagia and other eating and drinking difficulties are common in progressive neurological diseases. Mealtimes can become a major source of difficulty and anxiety for patients and their families. Decisions about eating, drinking and care can become challenging as disease progresses, and the person in question loses the capacity to participate in decisions about their own care. We sought to investigate how patients and their family members make decisions about their future care as their condition deteriorates, with a particular focus on mealtimes, eating and drinking.

**Methods:**

Longitudinal qualitative in-depth interviews were undertaken with patients and their family members (*N* = 29) across a range of disease groups, including: dementia, Parkinson’s Disease, Huntington’s Disease, Progressive Supranuclear Palsy, Motor Neurone Disease, Multiple Sclerosis. Patients had varying degrees of eating and drinking difficulties, and levels of decision-making capacity. Interviews were ‘participant led’ and undertaken in the patients’ own homes or a place of their choosing. Follow-up interviews were three months to one year later depending upon disease trajectory. Interviews were audio recorded and analysed in NVivo using a Thematic Analysis approach.

**Results:**

Twenty-nine participants were interviewed between 2015 and 2017. Two key themes emerged from the analysis: 1) *Health Literacy:* the extent to which patients and relatives appeared to know about the condition and its treatment. Patients and their family members varied in their ability to speak and communicate about their condition and prognosis. 2) *Planning style:* the extent to which participants appeared to value involvement in advance care-planning. Patients and their family members varied in the way in which they made decisions: some preferred to ‘take each day as it comes’, while others wished to plan extensively for the future.

**Conclusions:**

Issues with eating and drinking are often overlooked. Clinicians need to understand both the patient’s level of health literacy and their style of planning before communicating with patients and their families about these sensitive issues.

## Background

Mealtimes are usually an important part of everyday life, with opportunities for pleasure and socialising [1, 2]. Social meanings attached to mealtimes are connected to deep experiences of self, childhood, care and identity [2–4]. People with progressive neurological diseases experience complex and unpredictable changes in their physical, cognitive, emotional and behavioural functions which can affect their decision-making capacity and their abilities at mealtimes, including changes in swallowing ability. These vary between individuals and across disease groups [5–11].

Estimates of the prevalence of dysphagia range from: 13–57% in dementia [12]; 32–85% in Parkinson’s Disease (PD) [13]; 37% in Huntington’s Disease (HD) [14]; 30–100% in Motor Neurone Disease (MND) [15]; 34–81% in Multiple Sclerosis (MS) [16, 17]; and 16–80% in Progressive Supranuclear Palsy (PSP) [18–20]. Difficulties with eating and drinking may arise from: physical impairments (impaired neuromuscular coordination of swallowing, tremor, rigidity, impaired coordination); cognitive difficulties (not recognising food, forgetting to chew or swallow, forgetting mealtimes); and behavioural difficulties (refusing to chew or swallow, spitting out food, or mealtime aggression) [21–23]. These can cause weight-loss, malnutrition, dehydration and aspiration pneumonia [24]. Mealtimes can become a major source of difficulty and anxiety for patients and their families [25], with emotional distress from loss of selfhood, social isolation and fear of becoming a burden to others [26].

Pharmacological and non-drug interventions aim to improve patients’ quality of life through disease management and symptom control [27–30]. Support and interventions to address swallowing difficulties include; sitting with the individual at mealtimes, spooning food into the mouth, thickened food and drinks, nutritional supplements, and tube feeding by percutaneous endoscopic gastrostomy (PEG) or nasogastric (NG) tube [31, 32].

Tailoring these interventions to best suit the individual needs and wishes of each patient is an important part of the care-planning process. However, as illness progresses, decision-making capacity and communication ability may become impaired or lost, making shared decision-making more challenging. Early decisions are needed for some conditions, such as MND, as progression may be rapid with a short survival time and potential loss of decision-making capacity [10, 30]. For other conditions, such as dementia, PD and MS, the disease trajectory is typically longer, though unpredictable and uncertain, potentially allowing more time to make decisions [7, 8, 28]. If asked to make decisions in advance, patients are considering an uncertain and unknown future that may be difficult to contemplate at a time when they are adjusting to living with and coping with progressive and life-limiting illness.

### Aims

We therefore undertook a study of patients and their families with a range of progressive neurological diseases. We investigated their experiences and views on decision-making concerning their care as their disease progressed, with a focus on problems with eating and drinking. The key research question was:

How do patients and their family members make decisions about their future care, with a particular focus on mealtimes, eating and drinking?

## Methods

### Design and recruitment

Longitudinal qualitative interviews were used to collect data from people with progressive neurological disease, their carers and healthcare professionals. A purposive sampling strategy was used to recruit a maximum variety sample of participants by disease group, decision-making capacity and degrees of eating and drinking difficulties.

Patient ‘clusters’ were recruited comprising up to four participants: the patient (if they had the decision-making capacity to consent to participate); a friend or relative (“informal carer”); a healthcare professional; and a paid carer (if they had one). In the first instance, (Time one - T1) in-depth qualitative interviews lasting 20 to 90 min were conducted with each participant. The length of the interview was determined by each participant’s health and their responses to the questions. At a second optional ‘mealtime interview’ approximately one week later; the patient, relative and interviewer shared a meal in the participant’s home. Subsequent follow-up interviews (Time two - T2), were undertaken three to twelve months later depending upon the trajectory of the disease.

Clinical collaborators initially approached potential patient participants with a study pack containing: a cover letter, an information sheet, a reply slip and a ‘freepost’ return envelope. Upon receipt of a reply slip, a member of the study team arranged a meeting at which the study was further explained, decision-making capacity was assessed, and if the participant chose to participate, informed consent was obtained. Patient participants were invited to nominate a relative or friend, a healthcare professional and a paid carer, who were all approached to participate.

### Ethical issues

Capacity to consent to participation was assessed before each interview by the interviewer, who had received training in assessing decision-making capacity. Participants with capacity gave written consent, or verbal audio-recorded consent if they had communication or physical difficulties. If capacity was in doubt, a consultee was asked to provide written informed assent in accordance with the Mental Capacity Act 2005.

To minimise the potential for distress during discussion of possible future feeding difficulties, clinical collaborators only approached patients they judged suitable for such discussion and raised the research topic at that time. Further details were included in the participant information sheet. The interview schedule was participant-led; although the interviewer raised eating and drinking and deterioration in the condition in broad terms; these topics were only discussed in more detail if raised by participants. For example, PEG was only discussed if participants had already mentioned tube feeding. Participants were given contact details for support, including their GP, district nurse, specialist nurse, consultant and condition-relevant support groups or networks. None requested that the researcher contact these on their behalf. Ethical approval was given by the London South East Research Ethics Committee (REC reference: 14/LO/1156, IRAS project ID: 156054).

### Research sample

Forty-two participants were interviewed between one to three times each between January 2015 and April 2017. This paper focuses on the findings from the interviews with patients and their relatives (*N* = 29). Disease groups represented were Parkinson’s Disease (PD), Huntington’s Disease (HD), Motor Neurone Disease (MND), Progressive Supranuclear Palsy (PSP), Corticobasal Degeneration (CBD), Multiple Sclerosis (MS) and dementia (including both Alzheimer’s Disease and Frontotemporal Dementia). **(**Table 1**)** Gender identifiers have been removed from the text for reasons of confidentiality.

**Table 1:** Cross-tabulation of participants, disease groups and decision-making capacity (*N* = 29 participants consented)

Cluster code	Primary participant disease group	Primary participant decision-making capacity^a^	Primary participant age group in years	Years since diagnosis	Nominated relative or friend	Total no. of patients and relatives within cluster	No. of participants consented for interview^b^
A	Frontotemporal Dementia	Partial	65–80	6	Wife	2	2
B	Alzheimer’s Disease	No	65–80	3	Friend	2	1
C	Parkinson’s Disease	Yes	65–80	2	Partner	2	2
D	Parkinson’s Disease	Yes	65–80	1	Daughter	2	2
E	Multiple Sclerosis	Yes	40–64	19	N/A	1	1
F	Multiple Sclerosis	Yes	40–64	8	Husband	2	2
G	Motor Neurone Disease	Yes	65–80	< 1	Wife	2	2
H	Motor Neurone Disease	No	65–80	2	Husband	2	1
I	Motor Neurone Disease	Yes	40–64	2	N/A	1	1
J	Progressive Supranuclear Palsy	Yes	65–80	5	[Relative relationship redacted]	2	2
K	Huntington’s Disease	No	65–80	3	Husband	2	1
L	Huntington’s Disease	Yes	40–64	15	Husband	2	2
M	Multiple Sclerosis	No	65–80	23	Wife	2	1
N	Progressive Supranuclear Palsy	Partial	65–80	1	Husband	2	2
0	Motor Neurone Disease	Yes	65–80	< 1	Wife	2	2
P	Progressive Supranuclear Palsy	Yes	40–65	3	Husband	2	2
Q	Alzheimer’s Disease	No	65–80	12	Wife	2	1
R	Parkinson’s Disease	Yes	65–80	6	Husband	2	2
Totals						34	29

^a^Decision-making capacity assessed at time of interview by the interviewer in relation to the capacity to participate in a qualitative interview

^b^Where the primary participant (patient participant) had partial or variable decision-making capacity, assent for interview participation was taken from a consultee by the interviewer. Where the primary participant did not have the capacity to participate in an interview, interviews were not undertaken with them

### Data analysis

The interviews were audio-recorded, transcribed, anonymised and entered into NVivo 11 for analysis. Thematic analysis was used for data analysis and interpretation [33]. Coding was undertaken by GC, SaB and JT. Initial open inductive data driven coding was refined into broader themes that were further reviewed, refined and discussed between the coders to develop higher level analytical themes. Themes were refined on the basis of salience of content, as well as frequency of appearance.

## Results

Themes relating to the research question (‘what matters to patients and their carers when planning for the future, with a focus on eating and drinking?’) emerged in two groups: those relating to the extent to which patients and carers appeared to know about the condition and its treatment (*health literacy*), and those relating to the extent to which they appeared to value involvement in advance care-planning (*planning style*). These are presented in more detail below, with illustrative excerpts from the dataset.

### Theme one: Health literacy

Most participants characterised themselves as sufficiently informed about what may lie ahead for them:

**I:** *Are there any issues that you would like, or would have liked, more information on?*

**P:** *No, because at the time they told us there was nothing. So they were quite up front about that, weren’t they* [laughs] *and so everything plus has been a bonus ever since, hasn’t it?* [Carer of HD patient. **R622**].

**P:** *No, I think everyone’s been honest. Yes, even in terms of the timeframes you’ve got, I think everyone has been very honest, yes* [MS patient. **P412**]

However, this satisfaction was not universal. Some wanted more information on their condition and its likely progression, or about options for managing eating difficulties, particularly information about percutaneous endoscopic gastrostomy (PEG):

**P:** *If we’d have had more information then, we would have been more prepared for things as time goes on* [Carer of PD patient. **R223**].

**P:** *They could do a bit more to be progressive in trying to explain to people what they’re about to experience. It’s quite shocking if you, you know, you’re looking at it for the first time* [Carer of MND patient. **R322**].

**P:** *Well I think I would have probably gone for the PEG earlier, if I’d have known. Obviously when the PEG was going in I weren’t that sure because I didn’t really know about it then, but actually having it done has made my job a lot easier. I mean, it was difficult feeding him/her before that, you know* [Carer of HD patient. **R622**].

Participants’ ability to speak about their condition and potential future progression varied by disease group. Knowledge of prognosis appeared greatest in the HD, MND and PSP/CBD groups, with those in the Dementia and PD groups seemingly less able to articulate what lay in store.

#### Experts by experience

The HD group felt they had good knowledge of the condition because they had seen how it affected family members. They characterised this experience, rather than anything they had been told by healthcare professionals, as the source of their health literacy:

**I:** *And at that point did they explain to you how the illness would progress?*

**P:** *Well I already knew that from his/her mother and his/her aunties and uncles and it was, you know, family members really.*

**I:** *So they didn’t need to explain anything...*

**P:** *Not really, no.*

**I:** *Did they talk about issues with eating and drinking or is that something that you...?*

**P:** *Well no, because you didn’t really see that bit, you know, you didn’t really see that part of the relatives, you used to go and visit them but you didn’t really consider that and his/her aunty didn’t want a PEG, s/he stipulated that s/he didn’t want a PEG and obviously s/he had swallowing problems and died.*

**I:** *Yeah. So his/her aunty knew early on but s/he just didn’t want...?*

**P:** *Yeah, s/he didn’t want nothing like that, no, s/he didn’t want to be messed about* [Carer of HD patient. **R622**].

**I:** *So, at the time s/he was first diagnosed, did anyone talk to you about how the condition would progress?*

**P:** *We were quite aware of that … no, we haven’t really been told by anyone* [Carer of HD patient. **R621**].

#### Well informed from the outset

Participants in the MND and PSP/CBD groups spoke confidently and fluently about prognosis, often using medical vocabulary. They identified the information provided by health professionals as the source of their health literacy:

**I:** *…and did they speak to you about how the condition would progress?*

**P:** *Yeah. I mean that’s obviously part of the condition and that was always one of the things that* [Consultant] and [Consultant 2] *talked about in terms of the ability to swallow through the deterioration with the muscles. That was one of their biggest concerns about, obviously which would cause aspirational pneumonia, so they were very adamant about, you know, making sure that we did everything possible to ensure that we didn’t cause that. And they spoke obviously about the PEG and, you know, the benefits of the PEG* [Carer of CBD patient. **R523**].

**I:** *So when you saw the doctors at* [Clinic] *did they outline for you how the disease would progress?*

**P:** *Yeah. Yeah, I mean you get the kind of assessment that you would expect from a Consultant, and of course they have a Care Team there which you may be familiar with, so the moment they begin to think about something as serious as Motor Neurone, or whatever else it is, the Care Team are involved and they of course, they’re made up of a dietician, a speech therapist … there’s three of them, and you know, they go through the whole business in great detail. So, you know, you can’t fail to understand what’s going on and the support was very good at [Hospital] seen very regularly* [Carer of MND patient. **R322**].

**I:** *At that time, were you told about the progression of his/her condition?*

**P:** *Yeah, yeah, straight away he told us how, how it does progress but this seems to be progressing in a lot of ways different to the other people we see at the meetings, yeah? S/he still walks, most of the people we know are in wheelchairs and that, and s/he gets on quite well* [Carer of PSP patient. **R521**].

However, being ‘well informed’ by clinicians did not predict the participants’ choices. For example, within the group of four participants diagnosed with MND, all four had discussed PEG with their care team but: one chose PEG feeding; one was strongly considering PEG feeding but died unexpectedly during the course of the study, and two were early stage and undecided.

#### Mixed picture

In MS clusters, responses were more mixed. Some, much like the MND and PSP/CBD groups, appeared to have been kept fully informed by healthcare professionals:

**I:** *At that point, did they explain to you how the disease would progress?*

**P:** *Yes, it was, progressing was quite rapidly, and, but they did explain the care needs for the future, and what would be needed. Yes, yes, they did explain it quite harshly, and that’s what I want, you know, they did explain it fully* [MS patient. **P412**].

Others reported receiving little information from healthcare professionals, but were able to use other sources to develop their health literacy:

**I:** *So who first told you that you may or may not get, swallowing disorders in the first place?*

**P:** *Well, it’s part of the progression of the disease.*

**I:** *And when did they first raise that with you? Was that at the time of your diagnosis or was that later on?*

**P:** *Nothing was given to me at the time of diagnosis, it’s all been picked up as it’s gone along. Various online chat rooms about MS, talking to other people with MS, common sense on these things and also I always have to treat my MS as something I’m going to look forward in the future dealing with, rather than leaving it, how you deal with it on a catch-up basis so it’s best to be informed* [MS patient. P411].

However, others in the MS group described an apparent choice to remain uninformed, although information was available:

**I:** *And at that point did any healthcare professionals talk to you about how the disease would progress?*

**P:** *The information was there,* [Patient] *chose not to want to know really, s/he... I think it took her/him ten years to take it in, and that was her/him. I obviously looked up what was going to happen and had much more of an idea but s/he chose to not know* [Carer of MS patient. R423].

#### Still unaware

In contrast, participants in the dementia and PD clusters were less able to articulate their prognosis, rarely used technical language, and were sometimes hesitant in speaking. Unlike those in the MS group, who described choosing not to find out what lay in store; these participants appeared to have been offered less information than those in the other groups:

**I:** *And Dr* [Consultant] *has explained how the condition will evolve in the future?*

**P:** *Um, not really, I haven’t really asked. At least I did ask, you know, I know that s/he’s on the lower scale of dementia, sort of, you know, not up here but down here. I’ve no idea, I think it’s different for... I don’t think they can say. I think that’s why I haven’t been told, they can’t really say … Because, you know, I’m not sure if s/he will lose his mobility or if s/he will become rigid or shaky or you know, like Parkinson’s or something like that, I don’t know* [Carer of dementia patient. **R121**].

**I:** *So when you were there* [Clinic]*, did they talk about how the dementia would progress? Or the eating and drinking would either?*

**P:** *No. Nothing* [Lay carer dementia patient. **R122**].

**I:** *So has he talked to you about how the condition will progress in the future? Has the doctor explained……?*

**P:** *No. As far as he is concerned he doesn’t have an appointment with me for another six months* [PD patient. **P211**].

**I:** *And when they told you about that, did they tell you how about it might develop in the future?*

**P:** *No, not, it might not, it might just stay like it, so you know, I just carry on, and luckily it’s me left hand, me left arm, so I’m right-handed so it doesn’t affect me, it doesn’t keep me awake at night or nothing* [PD patient. **P212**].

### Theme two: Planning style

The extent to which participants expressed a wish to be involved in advance care-planning varied within and between the condition groups. Two main themes emerged: those who wanted to plan ahead and make their own decisions about care and treatment in advance, and those who preferred to live in the present, deferring decisions about future care. Some participants described using a mixture of those two approaches (with some switching preference as the condition progressed). For a small group of participants, the concept of care planning held little meaning as they did not perceive that there were any decisions to make.

#### ‘Advance planning’

Advance planning was used by some participants as a way of extending the zone of personal autonomy and involvement in decision-making beyond the stage when their ability to make decisions or communicate their wishes would be lost:

**P:** *In the early days of the diagnosis we obviously discussed his/her thoughts, his/her wishes going forward, s/he made her end of life wishes well known, they’re documented. I think I understand everything that* [Name] *wants and I totally agree with some of the things that s/he wants but yes, we have discussed that* [Carer of CBD patient. **R523**].

This group described a need for information about the kinds of interventions to support nutritional intake that might be considered as the condition progressed:

**P:** *We needed it* [information on PEG] *as far as I’m concerned, I mean it’s all bad news….you can mope around but there is the practical side to the whole business, even if it’s your wife or husband, you need to know what, what’s about to happen and what can be done* [Carer of MND patient. **R322**].

Some participants expressed a desire to be more engaged in the planning of care and were frustrated by the lack of information on offer:

**P:** *That’s where it becomes quite frustrating ‘cos I’m doing my utmost to do the best for the wife, naturally, and there doesn’t seem to be that outside help to guide you and put you on a little bit further and with a bit more help* [Carer of PD patient. **R223**].

However, the relationship between adoption of an ‘advance planning’ strategy and the need and desire for information was not straightforward. Some participants acknowledged a need for information but nonetheless did not want to be told everything:

**P:** *We got information mainly at* [Hospital] *but it was very sort of top level and; ‘Here’s some information but we don’t have to talk about it yet, you know, we’ll talk about it when you’re ready and unless we have to talk about it before’. So it was handed to me, for me to decide when I wanted to talk about it…*.. *I think we needed to know what lay ahead, but only at top level, and not to go into too much detail about anything that didn’t have an immediate or a near impact on me, certainly in the earlier days when you’re taking everything on board* [MND patient. **P313**].

Furthermore, information from health professionals did not always seem to be an important factor in the advance planning process. Some participants made all their own care decisions in advance, in keeping with their personality and based on long-term values:

**P:** *I mean that’s the way* [Name] *was, it may not be everybody’s way of doing things, but you know, as I said to you, s/he was very independent and s/he made his/her own decisions … so you know, s/he’s not the sort of person that’s going to, s/he’s going to rely on specialists to help him/her but s/he will be making his/her own decisions* [Carer of MND patient. **R322**].

These decisions were not always consistent with the advice and information offered by healthcare professionals. One participant planned to seek assisted suicide. Despite receiving information about options to alleviate their concerns about a distressing death, they chose to end their own life before the condition’s natural conclusion:

**I:** *So is there something particular that you’re frightened of in the progression, that makes you think you would like to end your life sooner? Or is there another reason you decided that you wanted to make the visit to Dignitas?*

**P:** *I don’t know because I spoke to* [Consultant] *last time about it. I was frightened of drowning in my own secretions, of choking to death. And he said it wasn’t like that, people usually slip away but, so, but I’m quite pragmatic about it because of this condition isn’t going to get any better. So I can’t see why prolonging it.*

[Patient identity withheld for reasons of confidentiality].

For this participant, reassurance that the dysphagia that they feared most would not arise did not have any impact upon the decision to seek assisted suicide. The provision of additional information to correct a possible misunderstanding did not change the decision. The inference that the decision was actually grounded in deep-seated values, rather than anxiety based on a potentially mistaken belief about the way the condition would progress, was expressed by their relative:

**P:** S*/he’s very practical, s/he’ll see it as the best thing all round for everybody, including her/himself; s/he said ‘I’m fed up with being like this’. S/he’s always sort of had her/his independence, s/he’s always worked, you know, had a good job* [Carer of patient who chose assisted dying; identity withheld for reasons of confidentiality].

#### ‘Take each day as it comes’

Some participants were hesitant to engage with, or discuss, decisions concerning their future care. Particularly in the early stages of illness they preferred to focus on the ‘here and now’, rather than thinking about problems that might lie ahead. Decisions about future care were deferred to a later date, sometimes in the apparent hope that the eventualities that they could plan for may never arise:

**I:** *Did they discuss about how it* [PD] *might develop in the future?*

**P:** *No, not, it might not, it might just stay like it, so you know, I just carry on* [PD patient. **P212**].

**P:** *So, I mean, that’s going to be an ongoing problem now I suppose? Unless something happens in... I don’t know. I don’t know if that happens in the brain, that things stop functioning and they start up again?* [Carer of Dementia patient. **R121**].

Some participants appeared to be aware of their prognosis, and of their developing problems with eating and drinking, but preferred not to focus on them. Sometimes this coping strategy was characterised as an active decision:

**P:** *I work out how to cope with it; don’t go thinking too much in the future. But of course we all have people like Stephen Hawking in our minds, and think, ‘is that where we’re going to end up, looking like him?’…. ‘And if that’s what people visualise us as, where we’re going to end up, it’s a bit of a daunting prospect’* [MND patient. **P314**].

Others discussed their swallowing difficulties in a way that suggested they were aware of the problem but, perhaps unconsciously minimising or attempting to ignore it:

**P:** *When I’m very tired I have to be more careful about my swallowing, and make sure that I chew my food thoroughly, but at the moment it doesn’t really affect me greatly … I do have episodes of choking when I’m drinking but other than that, no, it’s not a problem* [MS patient. **P411**].

Finally, some participants characterised their reluctance to be involved in future care planning as normal or expected for people of an older generation:

**I:** *So…. a will, but no plans about his/her care?*

**P:** *No, s/he said s/he knew I’d look after him/her, so I just left it at that. I don’t think people of that age like to talk about that, you know, it wasn’t done then, was it? It just happened* [Carer of Dementia patient. **R122**].

For participants who did not wish to engage in planning for the future, information about prognosis was not always welcome. Some wished that the information about diagnosis and prognosis had never been given:

**P:** *I would have rather they hadn’t told* [*patient*] *he had MND … If nobody told s/he’d got MND because s/he’d have just carried on thinking well, I’m getting old, I’ve got rheumatism, I can’t lift my arm, I can’t do this, can’t do that and s/he wouldn’t be stressed out and upset like s/he is, so* [Carer of MND patient. **R321**].

However, the relationship between this strategy and satisfaction with the amount of information provided was not straightforward. For some, it was information about prognosis that prompted them to choose this strategy:

**I:** *Did they talk to you then about how the illness would develop, or did they just sort of…….?*

**P:** *Well they did, a little bit, but in those days with Alzheimer’s Society, you know they don’t do it the same nowadays, there was a lovely lady … and she just said to me, she says* [Name], * “I’m going to be brutal”, and I said “Right, go on”, she said* “[Patient]*‘s fine now”, she says “Enjoy your time that you’ve got with him/her now because it will get worse and worse and worse”* [Carer of Dementia patient. **R123**].

For others, awareness of the complex and uncertain future lay behind their reluctance to engage in advance care planning. They expressed a preference for delaying important decisions until they had to be made, because it would be too difficult to make them without contextual information:

**P:** *I’d have to think about it at the time, because that means I wouldn’t want someone to cure me of pneumonia during a visit to A&E, if it’s just one of a succession of weekly visits to A&E, no, I wouldn’t want to be cured of pneumonia. But again, a person’s wishes should be taken into account* [PD patient. **P211**].

**P:** *No, I was asked about that* [making advance care decisions] *and I didn’t want to do it. I really don’t know how you can decide something like that* [MS patient. **P411**].

#### Mixed strategies

Participants did not always use a single coping strategy. As their disease progressed, some moved between ‘taking each day as it comes’ and ‘advance planning’.

**P:** *Well I did contact the MND Association up in* [Place], *I said, “Look, send me your pack and I’ll have a read,” and it was page after page, and as I explained to a doctor at* [Hospital], *it can be quite overwhelming, and he said, “Well that’s typical of charitable organisations, that they sort of paint a blacker picture, or tell you everything and you have to stand back and say, well is that relevant to me today, if that’s in the future we’ll worry about it in the future,” and this is how I think my wife and I have sort of come to terms with it, let’s live a day at a time, and we’ll navigate through sticky patches when we come to them* [MND patient. **P314**].

An added complexity was that patients and their carers sometimes adopted different strategies:

**P:** *I’ve started the Power of Attorney process but it’s with my husband now to finish it off, he’s a little bit of an ostrich, I think that’s fair to say* [MND patient. **R313**].

**P:** *Ah, me personally, yes, I would have done* [made advance care plans], *if that had happened to me I would have definitely chosen to do that, I don’t think* [Name] *is the type of person that would have done unfortunately* [Carer of MS patient. **R423**].

Some participants expressed regret that they had not engaged with care planning earlier in the course of their condition. For decisions concerning PEG feeding, this lack of advance planning appeared to be connected with a lack of information about the options. Two participants had PEGs fitted at a late, or crisis stage, and both of their carers wished they had made decisions earlier:

**I:** *So do you wish you’d had yours* [PEG tube] *fitted a bit earlier to stop those?*

**P:** *Yeah, it’s still a frightening thing but it could have been done in an easier time for his/her body* [Carer of PSP patient. **R521**].

**P**: *It was in the hospital, yeah, we made the decision. S/he went in, s/he had an abscess on his/her lung where s/he’d been swallowing food down the wrong hole … well basically s/he nearly died but the antibiotics kicked in and s/he was okay and then we had the PEG fitted in there, we had a lot of swallow tests and that done and then it was decided that the only way really was a PEG…*. *If I’d have known and s/he would have known I think we’d have had it a lot earlier* [Carer of HD patient. **R622**].

#### No decision to be made

For some participants the concept of care planning did not make any sense, as they viewed the progression of their condition, and the need to accept increasingly invasive treatment in order to stay alive, as part of an inexorable process. Consequently, there was no sense that they were choosing between planning in advance, or living in the present and delaying making decisions about care until they could be put off no longer. These participants described just doing what it took to survive:

**I:** *So is thickening your food and drink something that you would consider, despite the horrible taste?*

**P:** *Well, I’ll do that when I’ve got no choice really.*

**I:** *And what about PEG feeding?*

**P:** *It might be the only way to feed me* [MS patient. **P411**].

## Discussion

These results have clear implications for health professionals attempting to deliver person-centred care for people affected by progressive neurological conditions. One of the most striking findings is the variation, between diagnostic groups, in the degree to which participants appeared to be aware of what the future may hold for them, and the treatment options that may be available. It is unclear why those in the Parkinson’s disease and dementia groups were, in comparison with those affected by other conditions, less able to describe their prognosis. It may be that they had been provided with information but were unable to recall it at the time of the interview, perhaps as a result of cognitive impairment. However, it is possible that health professionals had decided to provide the most detailed information about prognosis, including the possibility of developing dysphagia, to people with MND, PSP and CBD, as these are conditions with a relatively rapid progression and a high likelihood of developing dysphagia. Thus, patients with these conditions may be seen as having a greater need for information as they are more likely to be faced with a need to make a decision about tube feeding in the near future. In contrast, patients with dementia and Parkinson’s disease, which have a slow and uncertain trajectory, may be perceived as less likely to have to make such a decision. The potential risks of giving them information about treatment for a distressing symptom that they may never develop may therefore appear to outweigh the potential advantage of enabling them to be involved in the decision to use tube-feeding, should this decision need to be made in the future after they have lost decision-making capacity. This approach could be characterised as ‘physician-centred’: the physician makes a decision about the patient’s information needs, based on his or her expert knowledge of the natural history of the patient’s condition and the likelihood of particular treatments becoming appropriate in the foreseeable future.

However, the findings of this study suggest that this approach may not be the most effective way of meeting the needs of individual patients. An understanding of their attitude to advance care planning may provide a more useful guide. The majority of participants were divided into two groups: those who wanted to deal with healthcare issues as they arose, live for the moment and not think about the future; and those who asked questions of healthcare professionals, researched their condition on their own and wanted to participate in shared decision-making and advance planning. Furthermore, the degree of health literacy demonstrated in the interviews did not predict participants’ approach to care planning.

For both groups, their approach could be seen as a mechanism for coping with the emotional burden and magnitude of their diagnosis and its implications. Charmaz has suggested that the suffering of chronic illness involves the psychological suffering of the loss of selfhood. She argues that, as disease progresses, individuals may develop visible disabilities resulting in stigmatised identities, or may suffer from discreditation of their identity due to reduced participation in everyday life [26]. Being unable to participate in mealtimes or having visible problems with eating and drinking are such an example. Participants choosing a strategy of ‘taking each day as it comes’ can use this to resist stigmatisation, and maintain their self-image as a ‘healthy person’ for as long as possible. This avoids disrupting the narrative of their life which could be psychologically destructive to their sense of self, alongside the distress of the damage the illness is doing to their body. Conversely, engaging in active planning and taking control of the situation, may help the person to distance their own self-image, from the image of ‘a sick person’ who lacks autonomy and self-possession. Charmaz argues that the self-discreditation of the chronically ill occurs when individuals can no longer take for granted an attribute they view as fundamental. This could include the power to make one’s own decisions.

The information needs of the two groups were not straightforward. The group who preferred to ‘take each day as it comes’ sometimes chose to do so because of the information they had received regarding prognosis, and some who had not made advance plans concerning the use of PEG feeding came to regret not doing so. Conversely, not everyone who engaged in advance planning wished to know everything about the condition or its treatment, with some expressing a wish for healthcare professionals to filter the information provided, to avoid overwhelming them, and others taking information provided by healthcare professionals into account but ultimately basing their decisions on other factors. However, an understanding of the strategy that each individual patient is adopting could usefully guide healthcare professionals to engage in sensitive, nuanced conversations that provide their patients with the information that the need to know. Furthermore, the evidence that people may switch between groups as the condition progresses suggests a need for healthcare professionals to remain alert to this possibility and adjust accordingly.

### Potential limitations of the study

One limitation of the research design of this study is that it may have over-represented participants who were ‘planners’, as only participants who were judged willing to talk about the future were approached. Those who wanted to not think about their condition could be underrepresented: the population may include a larger number of individuals who are less engaged with healthcare and prefer not to discuss about their condition.

Some participants suggested their approach to the management of their own disease was a continuation of their personality. However, these approaches may just have been about the participants using the psychological and emotional tools available to them at the time, and not indicative of an underlying essential difference. In fact, some participants used a mixed strategy approach to their disease management, engaging at certain times and selectively ignoring symptoms at others.

## Conclusions

This longitudinal qualitative study of patients with progressive neurological disease and their families investigated planning for the future and decision-making concerning eating and drinking. The thematic analysis revealed two key themes: 1) *Health literacy:* the extent to which patients and carers appeared to know about the condition and its treatment; and 2) *Planning style:* the extent to which they appeared to value involvement in advance care-planning.

Issues around eating and drinking are often overlooked by doctors and seen as the remit of speech and language therapists and nurses. However, the findings from this study showed the key role of eating and drinking in care-planning and the need for all clinicians to understand both the patient’s level of health literacy and their style of planning for the future before communicating about these sensitive issues.

## Abbreviations

HD: Huntington’s Disease
ID: Identification/Identifier
IRAS: Integrated Research Approval System
MND: Motor Neurone Disease
MS: Multiple Sclerosis
NG: Nasogastric
PD: Parkinson’s Disease
PEG: Percutaneous Endoscopic Gastrostomy
PSP: Progressive Supranuclear Palsy
REC: Research Ethics Committee
T1: Time One
T2: Time Two
